# Staged limb salvage of a Gustilo–Anderson type IIIC open distal tibial gunshot fracture using cross-leg pedicle flap and delayed intramedullary nailing with bone grafting: a case report

**DOI:** 10.3389/fsurg.2026.1840063

**Published:** 2026-06-11

**Authors:** Abdikarim Mahad Ahmed, Osman Mohamed Abdulkarim, Abdijalil Abdullahi Ali, Mohamed Osman Dahir Alasow, Nur Adam Mohamed, Abdullahi Khalif Ali

**Affiliations:** 1 Department of Orthopedic and Traumatology, Mogadishu Somali-Turkiye Recep Tayyip Erdogan Training and Research Hospital, Mogadishu, Somalia; 2 Department of Cardiovascular Surgery, Mogadishu Somali-Turkiye Recep Tayyip Erdogan Training and Research Hospital, Mogadishu, Somalia; 3 Department of Radiology, Mogadishu Somali-Turkiye Recep Tayyip Erdogan Training and Research Hospital, Mogadishu, Somalia; 4 Department of Psychiatry, Mogadishu Somali-Turkiye Recep Tayyip Erdogan Training and Research Hospital, Mogadishu, Somalia; 5 Department of General Surgery, Mogadishu Somali-Turkiye Recep Tayyip Erdogan Training and Research Hospital, Mogadishu, Somalia

**Keywords:** bone graft, cross-leg pedicle flap, gunshot injury, Gustilo–Anderson type IIIC, intramedullary nailing, open tibial fracture, vascular injury

## Abstract

**Introduction and importance:**

Gunshot injuries to the lower limb can cause severe damage to both bone and soft tissues, particularly when the injury occurs at close range. Open fractures of the tibia are especially difficult to manage because of the limited soft-tissue coverage and the high risk of infection, non-union, and other complications. Gustilo–Anderson type IIIC fractures represent the most severe form, as they involve arterial injury that requires urgent repair. Successful limb salvage depends on early debridement, restoration of blood flow, stable fixation, and reliable soft-tissue coverage. In settings where advanced reconstructive options are not available, staged approaches may offer a practical alternative.

**Case presentation:**

A 25-year-old previously healthy man sustained a gunshot injury to the distal right leg, resulting in a severe open tibial fracture with extensive soft-tissue loss, segmental bone loss, and injury to the posterior tibial artery. He underwent urgent irrigation, thorough debridement, primary arterial repair, and temporary external fixation. The tibial nerve was preserved. Serial debridements were performed until the wound was clean and well perfused. Soft-tissue coverage was then achieved using a cross-leg pedicle flap, with pedicle division performed after three weeks. This was followed by delayed intramedullary nailing, supported by a fibular strut graft and autologous iliac crest bone grafting. At six months, imaging showed progressive healing, and the patient regained full weight-bearing ability with independent ambulation and good functional recovery.

**Clinical discussion:**

Managing severe open tibial fractures with vascular injury requires a carefully staged and multidisciplinary approach. Initial priorities include infection control and restoration of blood flow, followed by soft-tissue coverage and definitive skeletal reconstruction. Cross-leg pedicle flaps remain a reliable option when free-flap reconstruction is not feasible, particularly in resource-limited environments. In selected cases, staged bone reconstruction using grafts and stable fixation can provide satisfactory outcomes.

**Conclusion:**

A staged treatment strategy combining vascular repair, repeated debridement, cross-leg flap reconstruction, and delayed intramedullary fixation with bone grafting can achieve successful limb salvage in selected patients with severe open tibial gunshot injuries, especially in resource-constrained settings.

## Introduction

Gunshot injuries to the lower extremity may cause large-scale bone and soft-tissue destruction, particularly in close-range injuries (1, 2). Open tibial fractures are especially challenging because of the tibia's limited soft-tissue envelope and the high risk of contamination, infection, non-union, malunion, and soft-tissue complications (1–4). Severe open tibial fractures (Gustilo–Anderson type III) are associated with substantial morbidity and often require staged multidisciplinary management (2–4).

Gustilo–Anderson type IIIC open fractures represent the most severe subgroup and are defined by an associated arterial injury requiring vascular repair (4). Successful limb salvage depends on urgent debridement, restoration of perfusion, fracture stabilization, and durable soft-tissue coverage (4, 6).

Traditionally, open fractures with major bone and soft-tissue loss may be treated using serial debridement, temporary external fixation, soft-tissue reconstruction, and staged definitive fixation with or without structural grafting (5, 6). Free vascularized osteocutaneous reconstruction is a reliable option for selected patients with large bony defects, but it requires suitable recipient vessels and microsurgical expertise (7). When free-flap reconstruction is unavailable or relatively contraindicated—particularly in the setting of vascular injury—the cross-leg pedicle flap remains a valuable limb-salvage option (8, 9).

We report a case of a Gustilo–Anderson type IIIC open fracture of the distal tibia caused by a gunshot injury with vascular injury requiring posterior tibial artery repair and extensive bone and soft-tissue loss, treated with staged debridement, temporary external fixation, cross-leg pedicle flap coverage, and delayed intramedullary nailing with fibular graft augmentation and autologous iliac crest bone grafting.

## Case presentation

### Patient information

A 25-year-old previously healthy man sustained a gunshot injury to the distal right leg.

### Clinical findings

Clinical examination revealed a large open wound over the distal tibia with exposed bone and extensive soft-tissue loss (Figure 1A). Radiographs demonstrated a comminated distal tibial fracture with segmental bone loss and retained metallic fragments (Figure 1B).

**Figure 1:**
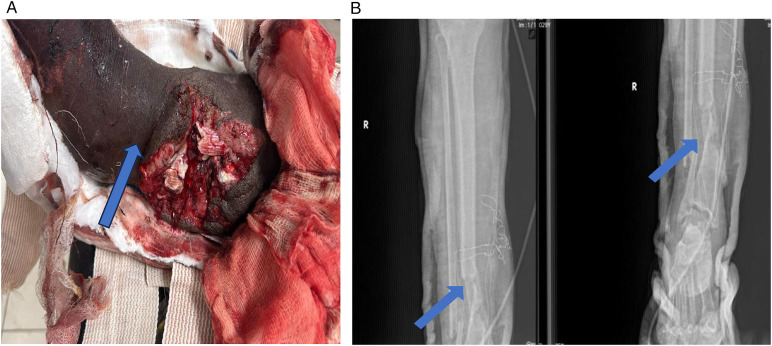
**(A)** Clinical photograph demonstrating an open distal right leg wound with exposed tibial bone following a gunshot injury. **(B)** Preoperative radiograph (anteroposterior view) demonstrating a comminuted displaced fracture of the distal right tibia and fibula.

Computed tomography angiography demonstrated vascular injury, and surgical exploration confirmed posterior tibial artery injury (Figures 2A,B). Distal limb perfusion was reduced but preserved. The anterior tibial artery was clinically non-palpable and likely compromised by the zone of injury. However, distal perfusion was maintained through collateral circulation, most likely from the peroneal artery, with retrograde flow contributing to the distal posterior tibial artery through communicating branches. Capillary refill was delayed but present, and handheld Doppler signals were weak but detectable. Perfusion improved following primary repair of the posterior tibial artery. The tibial nerve was found to be intact.

**Figure 2:**
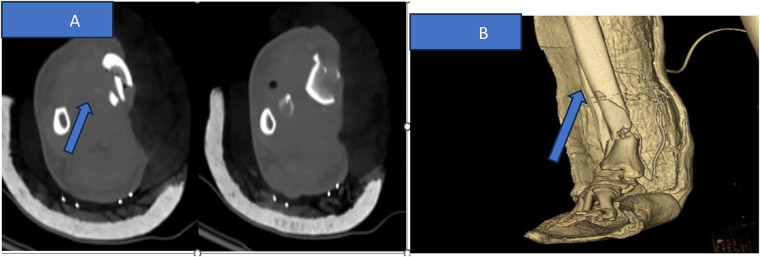
**(A)** Computed tomography angiography of the right leg demonstrating arterial injury with a segment of absent contrast opacification consistent with vascular disruption. **(B)** Three-dimensional reconstruction demonstrating the associated tibial fracture and vascular injury.

The injury was classified as a Gustilo–Anderson type IIIC open fracture.

### Therapeutic intervention

Initial management included urgent irrigation, radical debridement, posterior tibial artery repair, and temporary external fixation approximately 2 h after injury.

Serial debridements were performed on post-injury days 3, 6, 9, and 14 until a clean and well-perfused wound bed was achieved.

Due to extensive soft-tissue loss, definitive coverage was achieved using a cross-leg pedicle flap (Figures 3A,B). The flap was designed on the medial aspect of the contralateral leg as a pedicled fasciocutaneous flap including the deep fascia to preserve perforator blood supply. It measured approximately 16 × 8 cm, based on the dimensions of the defect. Preoperative Doppler mapping was not available; therefore, flap planning relied on anatomical landmarks corresponding to known perforator zones. The flap was carefully elevated and transposed to cover the distal tibial defect. Both limbs were immobilized using cross-leg external fixation to maintain position and prevent tension on the pedicle.

**Figure 3:**
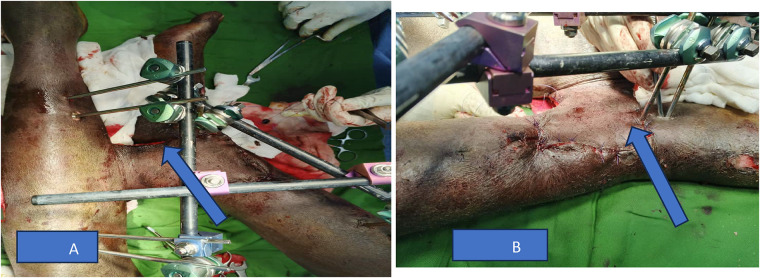
**(A)** Immediate postoperative appearance following cross-leg pedicle flap reconstruction demonstrating coverage of the anterior tibial soft tissue defect. **(B)** Donor site covered with a split-thickness skin graft.

After three weeks, pedicle division was performed and the external fixator was removed (Figures 4A,B). Partial weight-bearing was initiated thereafter.

**Figure 4:**
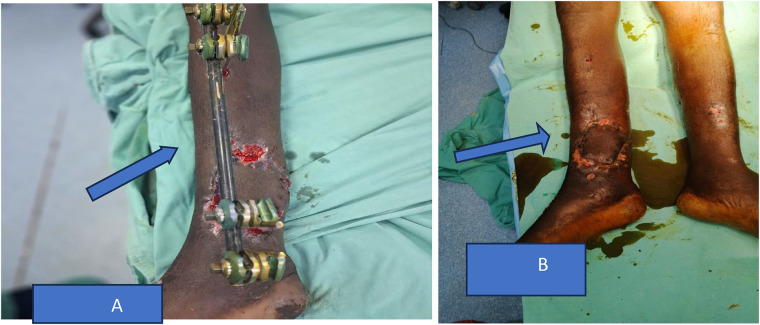
**(A,B)** Clinical photographs demonstrating cross-leg flap pedicle division performed three weeks after reconstruction, showing stable flap survival.

Following complete soft-tissue healing, delayed intramedullary nailing was performed. A non-vascularized fibular strut graft was used to provide structural support across the tibial defect and maintain alignment. The graft was positioned intramedullary within the defect and stabilized in conjunction with the intramedullary nail. Autologous cancellous bone graft harvested from the iliac crest was placed at the graft–host interfaces to enhance osteogenesis and promote biological union osteogenesis and promote biological union (Figures 5A,B).

**Figure 5:**
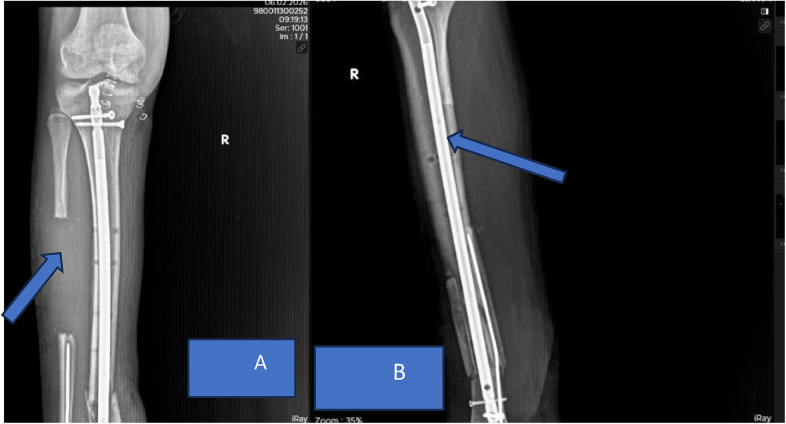
**(A)** Postoperative radiograph (AP view) demonstrating delayed intramedullary nailing with fibular strut graft and autologous iliac crest bone grafting, showing satisfactory alignment and implant positioning. **(B)** Lateral view confirming stable fixation, maintained fracture alignment, and adequate placement of bone graft material without implant-related complications.

No flap necrosis, surgical site infection, or implant-related complications were observed during the follow-up period.

Clinical photograph at follow-up demonstrating the patient ambulating with crutches. The cross-leg pedicle flap remains viable with satisfactory soft tissue coverage, preserved limb alignment, and progressive functional recovery (Figure 6).

**Figure 6:**
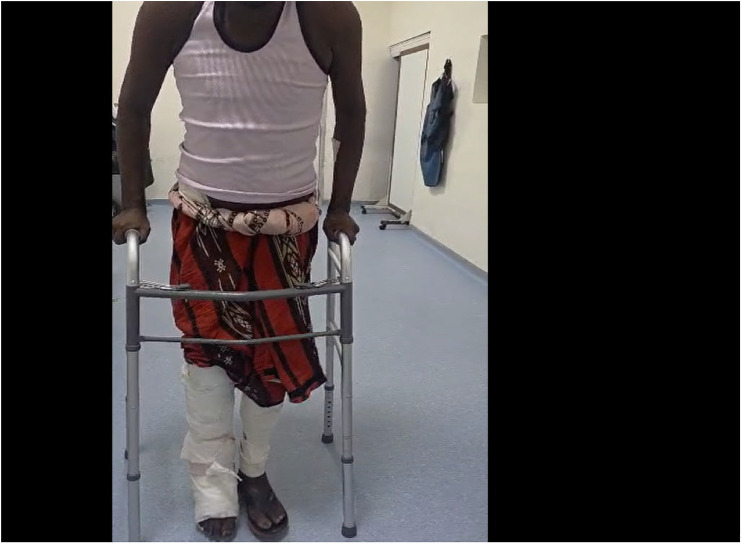
Clinical photograph at follow-up demonstrating the patient ambulating with crutches. The cross-leg pedicle flap remains viable with satisfactory soft tissue coverage, preserved limb alignment, and progressive functional recovery.

Validated functional outcome scores such as LEFS, SF-36, or AOFAS were not available for this patient and this is acknowledged as a limitation.

### Timeline of clinical course

Day 0: Injury, debridement, vascular repair, external fixation
Days 3, 6, 9, 14: Serial debridement
Week 3: Cross-leg pedicle flap
Week 6: Pedicle division and fixator removal
Delayed stage: Intramedullary nailing with bone graft
1 month after fixation: Full weight bearing
6 months: Follow-up

### Patient perspective

The patient expressed satisfaction with the outcome. Despite the inconvenience of cross-leg immobilization, he tolerated the procedure well and was pleased to retain his limb and regain functional mobility.

## Discussion

Management of open tibial fractures with segmental bone loss, extensive soft-tissue loss, and major vascular injury caused by gunshot trauma is highly challenging (2). Successful treatment requires infection control, restoration of perfusion, skeletal stabilization, reliable soft-tissue coverage, and staged reconstruction while minimizing complications (4–6).

Free vascularized osteocutaneous flaps such as fibular and iliac crest flaps are established options with satisfactory outcomes in selected patients (7). However, recipient-vessel integrity and microsurgical resources are essential for success. In our case, vascular injury requiring repair, together with a wide zone of trauma, reduced the feasibility of microsurgical free-flap reconstruction and supported a staged alternative approach (7–9).

Bone transport is an effective technique for long bone defects, but prolonged external fixation may cause joint stiffness, decreased quality of life, and pin-tract infection (5, 6). In selected cases, staged reconstruction using structural graft augmentation combined with stable fixation may provide a practical alternative (10).

Cross-leg pedicle flap reconstruction remains a historical yet dependable option for lower-extremity trauma when free-flap surgery is unavailable or relatively contraindicated (8, 9, 12). Its advantages include reliable flap survival, shorter operative time, and independence from the vascular condition of the injured limb (8, 9). Potential complications include joint stiffness and pressure-related problems; however, careful local hygiene, limb care, and rehabilitation may reduce these risks (12). The patient was able to perform basic daily activities with support and demonstrated progressive functional improvement.

Despite its advantages, the cross-leg pedicle flap has several limitations, including prolonged immobilization, patient discomfort, risk of joint stiffness, and challenges in postoperative care. Flap reliability remains a subject of discussion, particularly in patients with compromised vascular status. Some authors advocate a delayed flap elevation technique to enhance vascularity through the opening of choke anastomoses (13), which may improve flap survival. In the present case, immediate flap transfer was performed due to the urgency of soft-tissue coverage and resource limitations, and it resulted in successful flap survival without complications.

In this case, the choice of a cross-leg pedicle flap was influenced by the presence of vascular injury and the limited availability of microsurgical free-flap reconstruction. Alternative strategies such as the Masquelet technique, bone transport, and primary amputation were considered; however, a staged limb salvage approach was selected based on clinical feasibility and resource considerations.

In this patient, serial debridement and temporary stabilization were performed initially alongside vascular repair. Definitive soft-tissue coverage was achieved using a cross-leg pedicle flap, followed by delayed intramedullary nailing with fibular graft augmentation and autologous iliac crest bone grafting after flap maturation. This staged sequence prioritized infection control and durable soft-tissue coverage before internal fixation and contributed to satisfactory early functional recovery.

Quantitative parameters such as exact bone defect size, range of motion, and detailed functional scores were not available for this patient, which represents a limitation of this report. Radiological assessment was based on serial imaging demonstrating progressive healing; however, formal scoring systems such as the Radiographic Union Scale for Tibial fractures (RUST) were not applied. Additionally, detailed intraoperative vascular parameters such as ischemia time and postoperative vascular monitoring protocols were not fully documented, which is acknowledged as a limitation.

## Conclusion

This case highlights the value of staged management for a Gustilo–Anderson type IIIC open distal tibial gunshot fracture with vascular injury, extensive soft-tissue loss, and segmental bone loss. Serial debridement, temporary external fixation, vascular repair, cross-leg pedicle flap coverage, and delayed intramedullary nailing with fibular strut graft and autologous iliac crest bone grafting enabled successful limb salvage and satisfactory functional recovery without microsurgical free-flap reconstruction. This staged approach may be considered a feasible limb-salvage option in selected patients, particularly in resource-limited settings; however, conclusions are limited by the single-case design (8, 9, 12).

## Data Availability

The original contributions presented in the study are included in the article/Supplementary Material, further inquiries can be directed to the corresponding author/s.
